# Effects of Transcranial Direct Current Stimulation (tDCS) Over the Frontal Polar Area on Motor and Executive Functions in Parkinson’s Disease; A Pilot Study

**DOI:** 10.3389/fnagi.2018.00231

**Published:** 2018-07-30

**Authors:** Koji Ishikuro, Nobuhiro Dougu, Takamasa Nukui, Mamoru Yamamoto, Yuji Nakatsuji, Satoshi Kuroda, Isao Matsushita, Hiroshi Nishimaru, Mariana F. P. Araujo, Hisao Nishijo

**Affiliations:** ^1^Rehabilitation Department, Toyama University Hospital, Toyama, Japan; ^2^System Emotional Science, Graduate School of Medicine and Pharmaceutical Sciences, University of Toyama, Toyama, Japan; ^3^Department of Neuropathic Internal Medicine Neurology, Graduate School of Medicine and Pharmaceutical Sciences, University of Toyama, Toyama, Japan; ^4^Department of Neurosurgery, Graduate School of Medicine and Pharmaceutical Sciences, University of Toyama, Toyama, Japan; ^5^Graduate Program in Neuroengineering, Edmond and Lily Safra International Institute of Neuroscience, Santos Dumont Institute, Macaiba, Brazil

**Keywords:** Parkinson’s disease, tDCS, frontal polar area, rehabilitation, sensory motor function, executive function

## Abstract

Parkinson’s disease (PD) is a neurodegenerative disorder with motor and non-motor symptoms due to degeneration of dopaminergic neurons. The current pharmacological treatments induce complications associated with long-term use. However, current stimulation techniques for PD treatment, such as deep brain stimulation (DBS), are too invasive. In this context, non-invasive brain stimulation including transcranial direct current stimulation (tDCS) may be a safe and effective alternative treatment for PD. We previously reported that anodal tDCS over the frontal polar area (FPA) improved motor functions in heathy subjects. Therefore, in the present study, effects of tDCS over the FPA on motor and cognitive functions of PD patients were analyzed. Nine PD patients (3 men and 6 women) participated in this cross over study with three tDCS protocols; anodal, cathodal or sham tDCS over the FPA. Each tDCS protocol was applied for 1 week (5 times/week). Before and after each protocol, motor and cognitive functions of the patients were assessed using Unified PD Rating Scale [UPDRS (part III: motor examination)], Fugl Meyer Assessment set (FMA), Simple Test for Evaluating hand Function (STEF) and Trail Making Test A (TMT-A). The results indicated that anodal stimulation significantly decreased scores of motor disability in UPDRS-III compared with sham and cathodal stimulation, and significantly increased scores of motor functions in FMA compared with sham stimulation. Furthermore, anodal stimulation significantly decreased time to complete a motor task requiring high dexterity in STEF compared with those requiring low and medium levels of dexterity. In addition, anodal stimulation significantly decreased time to complete the TMT-A task, which requires executive functions, compared with sham stimulation. To the best of our knowledge, this is the first clinical research reporting that tDCS over the FPA successfully improved the motor and non-motor functions in PD patients. These findings suggest that tDCS over the FPA might be a useful alternative for the treatment of PD patients.

## Introduction

Parkinson’s disease (PD) is characterized by a progressive loss of dopaminergic neurons in the substantia nigra *pars compacta* (SNc) and/or ventral tegmental area (VTA; Alberico et al., 2015) with an increased risk of PD in the aging population (Abdullah et al., 2015). Dopaminergic dysfunction induces functional imbalance between the direct and indirect basal ganglia circuits, abnormal burstic and oscillatory activity in the cortico-basal ganglia circuit, or distorted competition between the direct pathway and hyperdirect and indirect pathways (Alexander et al., 1986; DeLong, 1990; DeLong and Wichmann, 2009; Nambu et al., 2015; Haber, 2016). Resultant abnormal activity in the cortico-striatal-thalamic pathways leads to the emergence of PD motor (resting tremor, bradykinesia, rigidity, postural instability) and non-motor symptoms (cognitive deficits, depression, orthostatic hypotension, etc.). Thus, PD patients display degradation in activity of daily living (ADL) and quality of life (QOL). Although basic medical treatment of PD is pharmacotherapy (especially levodopa), with long term treatment most patients gradually develop motor fluctuation and dyskinesia that may be more debilitating than the PD symptoms (Hauser, 2009). In this context, deep brain stimulation (DBS) techniques have been established as an alternative to treat PD and are reported to be effective to ameliorate motor and non-motor dysfunctions (Wichmann and DeLong, 2006). A less invasive stimulation technique, dorsal column spinal cord stimulation (SCS), has also been recently proposed as an alternative approach to treat the motor symptoms of PD, especially gait disturbances (Fuentes et al., 2009; Santana et al., 2014; Pinto de Souza et al., 2017). However, the high risk and cost associated with invasive neurosurgical procedures remains a major problem to be solved (Benabid et al., 2009).

Neuro-rehabilitative methods using non-invasive brain stimulation are currently being explored as a safer alternative that can modulate cortical excitability (Nitsche et al., 2003b; Russo et al., 2017). In particular, transcranial direct current stimulation (tDCS) is a relatively easy and safe method to modulate cortical polarization by applying low intensity current (1.0–2.0 mA) in the scalp. Anodal stimulation in tDCS increases cortical excitability, while cathodal stimulation decreases it (Liebetanz et al., 2002; Nitsche et al., 2003a). Recent studies suggest that tDCS combined with rehabilitation has long-term effects on symptom amelioration in several neurological disorders (Khedr et al., 2013; Flöel, 2014; Meinzer et al., 2016). In these studies, anodal tDCS is generally applied over the sensory-motor region and the cathode is placed over the opposite supraorbital region for improving motor affective functions (Nitsche et al., 2003a,b).

To the present date, clinical studies on tDCS to treat PD symptoms have focused on two stimulation sites: primary motor cortex (M1) and dorsolateral prefrontal cortex (DLPFC). These studies have reported that anodal tDCS of M1 improves PD motor symptoms (Fregni et al., 2006; Benninger et al., 2010; Kaski et al., 2014a,b; Valentino et al., 2014; Yotnuengnit et al., 2018), while anodal stimulation of DLPFC improves cognitive and executive functions (Boggio et al., 2006; Pereira et al., 2013; Doruk et al., 2014; Manenti et al., 2016). Since PD patients have concurrent motor and cognitive impairments, a tDCS protocol that can alleviate both types of symptoms would be an important alternative for PD therapy. In this context, studies to look for other stimulation sites may yield promising results.

We previously reported that in heathy subjects, hemodynamic activity of the anterior dorsomedial prefrontal cortex (aDMPFC), which corresponds to the frontal polar area (FPA), is correlated to the performance improvement rate in a motor task requiring high hand dexterity, and that anodal tDCS of the FPA improves the performance in this task (Ishikuro et al., 2014). Furthermore, previous studies reported that the FPA, including the aDMPFC, is activated when subjects learn new motor task(s) (Zysset et al., 2002; Floyer-Lea, 2004), and lesions in these areas delay motor learning (de Guise et al., 1999; Richer et al., 1999). In addition, the FPA, including the aDMPFC, projects to the DLPFC, involved in higher cognitive functions (Carmichael and Price, 1996; Petrides and Pandya, 2007; Orr et al., 2015), and high frequency repetitive transcranial magnetic stimulation (rTMS) of the DLPFC induces dopamine release in the caudate nucleus (Strafella et al., 2001). These findings suggest that the FPA is involved in both cognitive and motor functions. Therefore, we hypothesized that active tDCS applied over the FPA would improve motor functions as well as executive functions in PD patients. To test this hypothesis, cognitive and motor functions of PD patients were evaluated before and after three tDCS protocols over the FPA. Cognitive function was evaluated by the Trail Making Test A (TMT-A), a neuropsychological test that provides information on visual search, scanning, sequencing and speed of processing (Doruk et al., 2014), which are essential to predict PD patients’ ability to complete instrumental activities of daily living (Higginson et al., 2013). Motor evaluation was comprised by three tests: Unified PD Rating Scale [UPDRS (part III: motor examination)], Fugl Meyer Assessment set (FMA) and Simple Test for Evaluating hand Function (STEF). While UPDRS-III is one of the most widely used scale to assess motor disability in PD, FMA is used to evaluate body motricity and motor recovery. In addition, since previous results suggested the involvement of the FPA in a task requiring fine motor skills (Ishikuro et al., 2014), STEF was applied to specifically evaluate hand function and dexterity.

## Materials and Methods

### Subjects

Nine PD patients, three men and six women [mean age, 77.5 ± 4.8 years (mean ± SEM) (68–83 years); mean disease duration, 69.2 ± 30.7 months (11–108 months)] participated in this cross over study. Table 1 shows detailed patients’ characteristics. All subjects’ stages except patient C were mild PD classes (Yahr 1–2) and none had severe cognitive dysfunction nor depression. During the study (3 weeks), the subjects received no pharmacological medication for PD such as DOPA decarboxylase (aromatic L-amino acid decarboxylase) inhibitor, dopamine precursor (L-DOPA), catechol-O-methyl transferase (COMT) inhibitor, dopaminergic agonist, monoamine oxidase B (MAO-B) inhibitor, or anticholinergic agent. This study was carried out in accordance with the recommendations of the principles of the Declaration of Helsinki, the Ethical Guidelines for Clinical Studies from the Japanese Ministry of Health, Labour and Welfare. The protocol was approved by the Ethics Committee for Human Clinical Researches in University of Toyama. All subjects gave written informed consent in accordance with the Declaration of Helsinki.

**Table 1:** Baseline characteristics of the nine PD patients.

Patient ID	Disease duration (months)	Affected side	Yahr grade
A	84	Right	2
B	72	Right	1
C	48	Right	3
D	48	Right	2
E	108	Right	2
F	60	Right	2
G	96	Right	2
H	96	Right	2
I	11	Left	1

### Intervention Protocol (tDCS With Rehabilitation)

**Figure 1:**
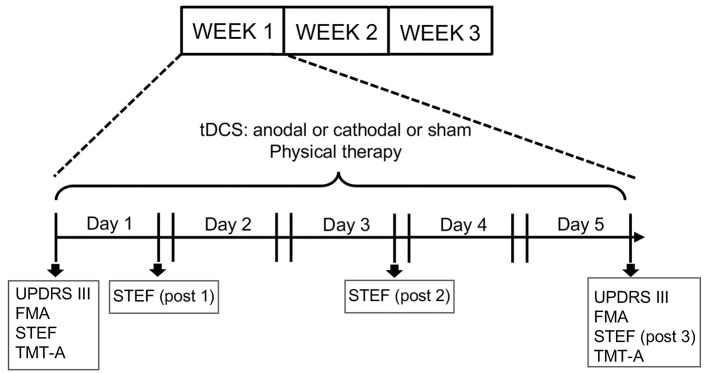
Experimental design of the present study. The experiments lasted 3 weeks. Each subject received three transcranial direct current stimulation (tDCS) protocols (Anodal, Cathodal and Sham stimulation) followed by physical therapy. Each protocol was applied for 5 days (1 week) in a randomized order. Unified PD Rating Scale (UPDRS-III), Fugl Meyer Assessment set (FMA) and Trail Making Test A (TMT-A) tests were applied every week before the first stimulation session (Day 1) and after the fifth session (Day 5), while the simple test for evaluating hand function (STEF) test was applied every week before the first stimulation session and after the first (post1), third (post 2) and fifth (post 3) stimulations sessions.

This clinical study employed a cross-over examination of three tDCS protocols (Anodal/Cathodal/Sham) and lasted 3 weeks. Each tDCS protocol was applied for 1 week (5 times/week: Monday–Friday) in a randomized order. On each experimental day, subjects received tDCS followed by physical therapy (Figure 1). The stimulation current was delivered by a battery-driven, constant current stimulator (DC-stimulator Plus, Neuroconn, Ilmenau, Germany) through a pair of saline-soaked sponge electrodes (5 × 7 cm) over the FPA and occipital area (OPA). These tDCS electrodes were placed on the head over the FPA and OPA according to the international 10–20 EEG system (Herwig et al., 2003). The tDCS conditions were same as those in our previous study with tDCS over the FPA using heathy subjects (Ishikuro et al., 2014); in the active tDCS protocols (anodal or cathodal stimulation), a constant current of 1.0 mA was delivered for 900 s, while 1.0 mA current was applied only for the initial 30 s (1/30 duration) in the sham tDCS protocol. The ramp up (down) was 1 s. The current density (0.0285 mA/cm^2^) was maintained below safety limits (Nitsche et al., 2003b; Poreisz et al., 2007). During the 3 tDCS protocols, the patients received traditional physical therapy in the upper extremities (stretching and muscle strength exercise) while sitting in a chair.

### Behavioral Assessment

#### Functional Assessment of Motor Symptoms

For motor assessments, the following tests were administered: UPDRS (part III: motor examination), FMA, and STEF. UPDRS part III assesses motor disability and includes ratings for tremor, slowness (bradykinesia), stiffness (rigidity) and balance (Louis et al., 1996; Martínez-Martín et al., 2000). Total possible score is 108 if all motor functions are fully disturbed. FMA is the reliability standard scale for measuring the sensory motor functions of patients after stroke and those with degenerative neuronal diseases including PD (Fugl-Meyer et al., 1975; Duncan et al., 1992). FMA provides assessment of sensory motor functions consisting of nine domains of sensory-motor functions (A: Shoulder; B: Wrist; C: Hand/Finger; D: Coordination in upper extremity; E: Hip/Knee/Ankle; F: Coordination in lower extremity, Balance; H: sensory; J: ROM/Pain). Total possible score is 226 if a subject has normal sensory-motor functions. UPDRS (part III) and FMA were measured twice (“pre” and “post”), where “pre” was defined as measures before stimulation, while “post” was defined as measures just after fifth stimulation (Figure 1). The data were normalized by “pre” measures (“post” measures/“pre” measures).

**Figure 2:**
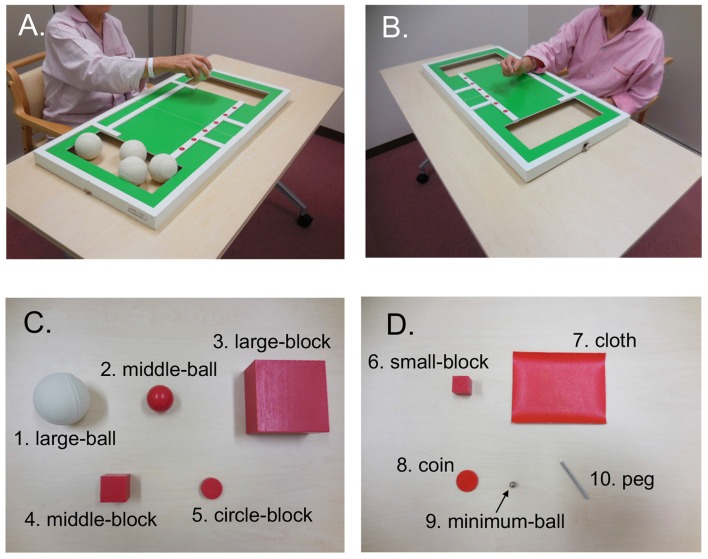
An illustration of the simple test for evaluating hand function (STEF). It was composed by 10 object-moving tasks using 10 different kinds of objects with different shapes and sizes. The patients were required to pick up these objects one by one from a storage space and move them into a target area as quickly as possible. **(A,B)** Photos of patients performing the large ball-moving **(A)** and peg-moving **(B)** tasks. **(C,D)** Objects used in STEF including large ball (1), middle-ball (2), large-block (3), middle-block (4), circle-block (5), small-block (6), cloth (7), coin (8), minimum-ball (9) and pegs (10).

The STEF (SAKAI Medical Co. Ltd., Hongo, Tokyo, Japan) has been developed and is used in Japan, and evaluates the patient’s ability to pinch, grasp, and transfer objects (Kaneko and Muraki, 1990). The test consisted of 10 object-moving tasks, requiring different levels of hand dexterity, that used objects with different shapes and sizes (large-balls, middle-balls, large-blocks, middle-blocks, circle-blocks, small-blocks, cloths, coins, minimum-balls and pegs; Figure 2). In each object-moving task, the patients were required to pick up one set of these objects one by one from a storage space and move them into a target area as quickly as possible. If the patient could not complete each object-moving task within a specific time limit (from 30 s to 70 s depending on the objects), the score of that task was 0. If the patient completed the task within the limit, the score was provided according to the time required to complete the task based on a pre-determined table for scores and time. The maximum score of each object-moving task was 10, and the total maximum score for the STEF was 100. Performance in STEF was measured four times (“pre”, “post1”, “post2” and “post3”), where “pre” was defined as measures before stimulation, and “post 1”, “post2” and “post3” were defined as measures just after first, third and fifth stimulation, respectively. The “post” data were normalized by “pre” measures (Figure 1).

#### Functional Assessment of Non-motor Symptoms (Executive Function)

For executive assessment, TMT-A was applied. TMT-A required patients to draw lines sequentially connecting 25 encircled numbers distributed on a test paper, and the time (sec) to connect from 1 to 25 was measured. TMT-A was applied twice (“pre” and “post”), where “pre” was defined as measures before stimulation, while “post” was defined as measures after fifth stimulation (Figure 1). The data were normalized by “pre” measures.

### Statistical Analyses

We analyzed the normalized scores in the motor [UPDRS (part III), FMA, and STEF (total score)] and non-motor tests (TMT-A) among the three tDCS protocols (Sham, Anodal and Cathodal) using one-way ANOVA (*post hoc*; Bonferroni test) or Kruskal-Wallis test (*post hoc*; Tukey’s range test), after a test for equal variances (Bartllet test).

In the STEF, the effects of tDCS on performance in tasks requiring different levels of hand dexterity was also analyzed. The normalized time to complete three different tasks (“large ball”-moving task requiring low level of hand dexterity, “circle block”-moving task requiring a medium level of hand dexterity, and “peg”-moving task requiring high level of hand dexterity) were analyzed by repeated measure two-way ANOVA (*post hoc*; Bonferroni test). Statistical significance was set at *P* value <0.05.

## Results

All nine patients completed the 3 weeks intervention protocol. During tDCS, five (55.6%) PD patients felt mild tingling. No other adverse effects were observed.

### Motor Functions

**Figure 3:**
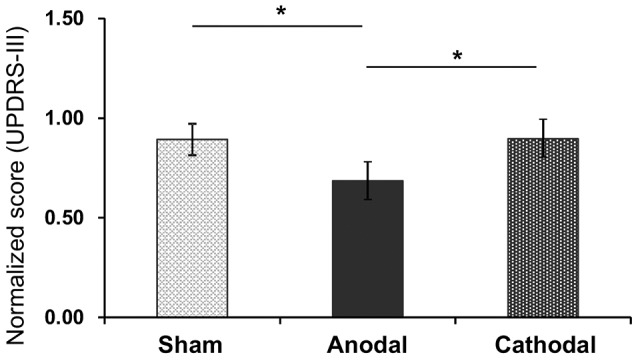
Mean normalized scores of motor disability in the UPDRS (part III) after each tDCS protocol (Sham, Anodal, Cathodal tDCS). The error bars represent the standard error of the mean (SEM). *p < 0.05.

The mean normalized scores of motor disability in the UPDRS (part III) after each tDCS protocol with rehabilitation are shown in Figure 3. The normalized scores were as follows; Sham (0.89 ± 0.08; mean ± SEM), Anodal (0.69 ± 0.15), and Cathodal (0.90 ± 0.18) tDCS. Comparison of the data by one-way ANOVA indicated a significant main effect (*F*_(2,26)_ = 6.484, *p* = 0.006). Multiple comparison tests indicated that Anodal stimulation significantly decreased normalized scores of motor disability compared with Sham and Cathodal stimulation (*p* < 0.05, Bonferroni test).

**Figure 4:**
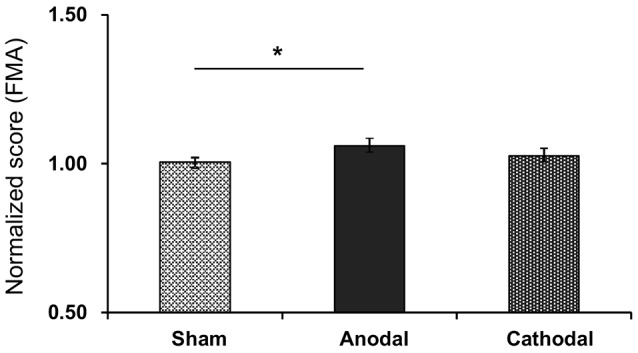
Mean normalized scores of sensory motor functions in the FMA after each tDCS protocol (Sham, Anodal, Cathodal tDCS). The error bars represent the SEM. *p < 0.05.

Figure 4 shows normalized scores of sensory motor functions in the FMA after each tDCS protocol with rehabilitation. The normalized scores were as follows; Sham (1.01 ± 0.02), Anodal (1.06 ± 0.06), and Cathodal (1.03 ± 0.03) tDCS. Comparison of the data by Kruskal-Wallis test after Bartlett test (Bartlett’s K-squared = 10.971, *p* = 0.0041) indicated a significant difference among the tDCS protocols (*H* = 6.719, *p* = 0.0348). *Post hoc* tests indicated that Anodal stimulation significantly increased normalized scores compared with Sham stimulation (*p* < 0.05, Tukey’s range test).

**Figure 5:**
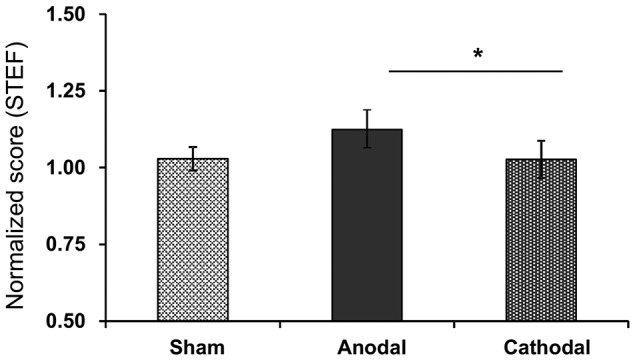
Mean normalized scores in the STEF after each tDCS protocol (Sham, Anodal, Cathodal tDCS). *p < 0.05.

The normalized scores of motor functions in the STEF after each tDCS protocol with rehabilitation were: Sham (1.03 ± 0.04), Anodal (1.12 ± 0.13), and Cathodal (1.03 ± 0.06) tDCS (Figure 5). Comparison of the data by Kruskal-Wallis test after Bartlett test (Bartlett’s K-squared = 11.453, *p* = 0.0033) indicated a significant difference among the tDCS protocols (*H* = 7.317, *p* = 0.026). *Post hoc* tests indicated that Anodal stimulation significantly increased normalized scores compared with Cathodal stimulation (*p* < 0.05, Tukey’s range test).

**Figure 6:**
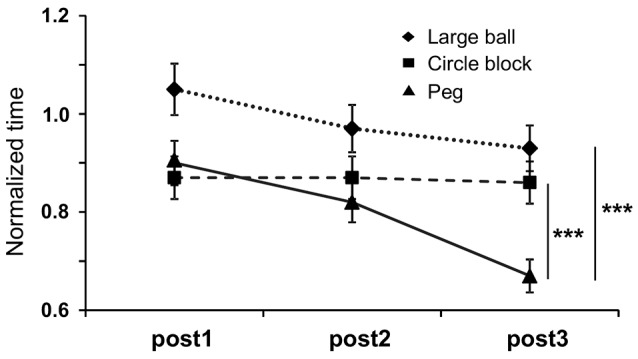
Mean normalized time to complete three different tasks requiring different levels of dexterity in the STEF; PEG-moving task (higher level of dexterity), circle block-moving task (medium level of dexterity) and large ball-moving task (low level of dexterity). “post 1”, “post2” and “post3” were defined as measures just after the first, third and fifth stimulations, respectively. The error bars represent the SEM. ***p < 0.0001.

The effect of Anodal tDCS on performance in STEF tasks requiring high (peg-moving task), medium (circle block-moving task) and low (large ball-moving task) levels of hand dexterity was also analyzed (Figure 6). A two-way repeated measures ANOVA with “task” (between-subjects factor) and “trial” (within-subjects factors) as factors indicated that there were significant main effects of “task” (*F*_(2,80)_ = 10.595, *p* = 0.0005) and “trial” (*F*_(2,80)_ = 8.369, *p* = 0.0008) and a significant interaction between “task” and “trial” (*F*_(4,80)_ = 2.635, *p* = 0.0454). *Post hoc* tests indicated that normalized time to complete the tasks was significantly lower in the peg-moving task than in both circle block- and large ball-moving tasks after the fifth stimulation (post3; *p* < 0.0001, Bonferroni test). In the Sham and Cathodal tDCS protocols, normalized time was analyzed in the same way. In the Sham tDCS protocol, the results indicated that there were no significant main effects of “task” (*F*_(2,80)_ = 0.089, *p* = 0.915) and “trial” (*F*_(2,80)_ = 0.964, *p* = 0.3885), nor significant interaction (*F*_(4,80)_ = 0.437, *p* = 0.7815). Furthermore, in the Cathodal tDCS protocol, there were no significant main effects of “task” (*F*_(2,80)_ = 0.892, *p* = 0.4231) and “trial” (*F*_(2,80)_ = 1.175, *p* = 0.3174), nor significant interaction (*F*_(4,80)_ = 1.602, *p* = 0.1891). These results indicated that the performance in the peg-moving task was improved after five consecutive days of Anodal tDCS.

### Non-motor Symptoms (Executive Function)

**Figure 7:**
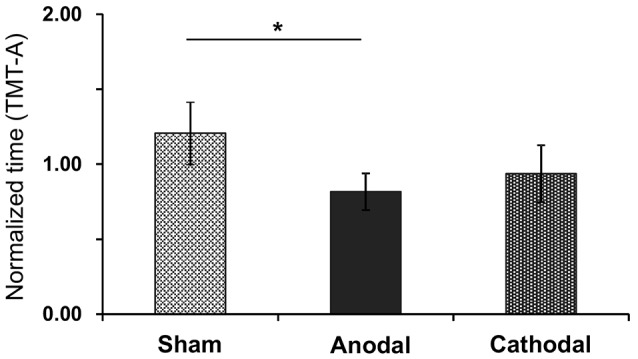
Mean normalized time in the TMT-A test after each tDCS protocol (Sham, Anodal, Cathodal tDCS). The error bars represent the SEM. *p < 0.05.

Figure 7 shows normalized time to complete the TMT-A test after each tDCS protocol with rehabilitation. The normalized time was as follows: Sham (1.21 ± 0.48), Anodal (0.82 ± 0.12), and Cathodal (0.94 ± 0.23) tDCS. Comparison of the data by Kruskal-Wallis test after Bartlett test (Bartlett’s K-squared = 12.9, *p* = 0.0016) indicated a significant difference among the stimulations (*H* = 7.801, *p* = 0.02), with *post hoc* tests indicating that Anodal stimulation significantly decreased normalized time compared with Sham stimulation (*p* < 0.05, Tukey’s range test).

## Discussion

In this study, we investigated whether tDCS over the FPA improved motor (UPDRS-III, FMA and STEF) and non-motor (TMT-A) functions in PD patients. The results indicate that anodal tDCS, but not sham or cathodal tDCS, significantly improved both motor and non-motor functions in PD patients. Moreover, our results suggest that the observed improvements were independent from physical therapy, since all groups received the same physical therapy treatment. To the best of our knowledge, this is the first clinical research reporting that tDCS over the FPA successfully improves both motor and non-motor functions in PD patients.

Motor improvements were observed in all three behavioral tests that were administered. Specifically, performance in the STEF tasks increased by 10.1%–34.1% after anodal tDCS compared with sham tDCS (Figures 5, 6), which is comparable to the previously reported improvement in the STEF task (21.9%) in healthy subjects after anodal tDCS over the FPA (Ishikuro et al., 2014). Importantly, in addition to the improvements in UPDRS-III and FMA scales, anodal tDCS specifically improved performance in the STEF task that required higher hand dexterity (peg-moving task) but not in the tasks requiring medium (circle block-moving task) and low (large ball-moving task) levels of hand dexterity (Figure 6). Previous studies also suggest that tDCS over M1 in PD patients improves gait and UPDRS-III scores (Fregni et al., 2006; Benninger et al., 2010; Kaski et al., 2014a,b; Valentino et al., 2014). The effects of tDCS over M1 on hand dexterity we so far less explored, with a previous report suggesting that it does not improve hand dexterity in PD patients (Fregni et al., 2006). It has been suggested that dexterity or fine motor control of the hand could be used to assess severity of PD (Pradhan et al., 2010; Dahdal et al., 2016). The present results along with these previous studies suggest that tDCS over the FPA could be useful alternative treatment of PD. In addition to motor improvements, tDCS over the FPA also improved cognitive functions, as measured by the TMT-A test. This result is similar to the ones described for tDCS over DLPFC (Boggio et al., 2006; Pereira et al., 2013; Doruk et al., 2014; Manenti et al., 2016). These previous studies, however, also indicate that tDCS over DLPFC is not effective in alleviating PD motor symptoms (Pereira et al., 2013; Doruk et al., 2014; Manenti et al., 2016). Therefore, the FPA may be an alternative site of stimulation to treat PD’s both motor and cognitive symptoms.

The neurophysiological mechanisms of tDCS over the FPA are yet to be determined. One plausible explanation would be that it may increase dopaminergic release from dopaminergic neurons in the VTA and SNc. The dopaminergic neurons in these areas receive direct and indirect glutamatergic projections from the PFC (Kalivas, 1993; Carr and Sesack, 2000; Omelchenko and Sesack, 2007; Han et al., 2017) and activity of the dopaminergic neurons is functionally coupled to the activity of PFC neurons (Gao et al., 2007; Zhang et al., 2012). In addition, inactivation of the PFC by cooling (Svensson and Tung, 1989), or by local injection of anesthetics (Murase et al., 1993) reduced burstic activity of dopaminergic neurons, while chemical and electrical stimulation of the PFC induced burstic activity of dopaminergic neurons (Murase et al., 1993; Tong et al., 1996b). Furthermore, the N-methyl-D-aspartate (NMDA) antagonist (3-((±)-2-carboxypiperazin-4-yl) propyl-l-phosphonic acid, CPP) blocked burstic activity of dopaminergic neurons during PFC stimulation (Tong et al., 1996a,b). Taking all these findings together, direct or indirect glutamatergic projections from the PFC to the midbrain are likely to be involved in burstic activity of dopaminergic neurons. In the present study, anodal tDCS over the FPA, which increased excitability of this area, improved motor and non-motor functions of the PD patients. The above inference suggests that tDCS over the FPA might increase burstic activity of dopaminergic neurons and subsequent dopaminergic release in the basal ganglia as well as the cortical areas, which might improve motor and non-motor functions in PD patients.

Although no formal safety guideline for tDCS procedure has been set to date (Nitsche et al., 2003a; Matsumoto and Ugawa, 2017), it has been reported that in a standard protocol (1–2 mA for 20 min stimulation with 25–35 cm^2^ large sponge), adverse effects are generally mild, and do not last after stimulation (Poreisz et al., 2007). The main adverse events previously observed were mild tingling (70.6%), moderate fatigue (35.3%), light itching (30.4%), etc. (Poreisz et al., 2007). Thus, tDCS is considered to be safe (Matsumoto and Ugawa, 2017). In the present study, 55.6% of the PD patients also felt mild tingling during tDCS. However, no other adverse events were observed. These findings suggest that tDCS over the FPA is a safe treatment that may be used on PD patients.

However, the present study has some limitations. First, the number of PD patients was small. Second, PD stage was relatively mild (Yahr 1–2). Although our results suggest that anodal tDCS over the FPA is useful in alleviating mild motor and cognitive symptoms, it is unknown whether tDCS is similarly effective to treat patients in severer PD stages. Third, each intervention period was relatively short (5 days). It is probable that tDCS with longer intervention periods would be more effective. Fourth, it is unknown whether dopaminergic functions in the midbrain changed or not after tDCS in the present study. Further studies with long term stimulation protocols will be required to investigate effects of tDCS over the FPA on midbrain dopaminergic neurons using non-invasive imaging techniques such as neuromelanin-MRI (Isaias et al., 2016) in PD patients. Fifth, long-lasting effects were not examined in the present study since we employed a cross-over examination of 3 tDCS protocols (Anodal/Cathodal/Sham). Further studies with a parallel-group comparison design are required to examine long-lasting effects of tDCS. Despite these limitations, this is the first study reporting that anodal tDCS on the FPA alleviates motor and cognitive symptoms in mild PD. These results are promising and provide the base for further studies on larger samples and with patients on different PD stages.

## Author Contributions

KI, IM and HisN designed research. KI, ND, TN, MY, YN and SK performed research. KI, HirN, MFPA and HisN analyzed data. KI, MFPA and HisN wrote the manuscript.

## Conflict of Interest Statement

The authors declare that the research was conducted in the absence of any commercial or financial relationships that could be construed as a potential conflict of interest.
